# Mutational spectrum and clinical features of *GBA1* variants in a Chinese cohort with Parkinson’s disease

**DOI:** 10.1038/s41531-023-00571-4

**Published:** 2023-09-01

**Authors:** Yangjie Zhou, Yige Wang, Juan Wan, Yuwen Zhao, Hongxu Pan, Qian Zeng, Xun Zhou, Runcheng He, Xiaoxia Zhou, Yaqin Xiang, Zhou Zhou, Bin Chen, Qiying Sun, Qian Xu, Jieqiong Tan, Lu Shen, Hong Jiang, Xinxiang Yan, Jinchen Li, Jifeng Guo, Beisha Tang, Heng Wu, Zhenhua Liu

**Affiliations:** 1 Department of Neurology, Xiangya Hospital, Central South University, Changsha, Hunan China; 2 Department of Neurology, & Multi-Omics Research Center for Brain Disorders, The First Affiliated Hospital, University of South China, Hengyang, Hunan China; 3 Department of Geriatrics, Xiangya Hospital, Central South University, Changsha, Hunan China; 4 Centre for Medical Genetics & Hunan Key Laboratory of Medical Genetics, School of Life Sciences, Central South University, Changsha, Hunan China; 5 Key Laboratory of Hunan Province in Neurodegenerative Disorders, Changsha, Hunan China; 6 Bioinformatics Center & National Clinical Research Centre for Geriatric Disorders, Xiangya Hospital, Central South University, Changsha, Hunan China; 7 Clinical Research Center for Immune-Related Encephalopathy of Hunan Province, Hengyang, Hunan China

**Keywords:** Parkinson's disease, Parkinson's disease, Genotype

## Abstract

*GBA1* variants are important risk factors for Parkinson’s disease (PD). Most studies assessing *GBA1*-related PD risk have been performed in European-derived populations. Although the coding region of the *GBA1* gene in the Chinese population has been analyzed, the sample sizes were not adequate. In this study, we aimed to investigate *GBA1* variants in a large Chinese cohort of patients with PD and healthy control and explore the associated clinical characteristics. *GBA1* variants in 4034 patients and 2931 control participants were investigated using whole-exome and whole-genome sequencing. The clinical features of patients were evaluated using several scales. Regression analysis, chi-square, and Fisher exact tests were used to analyze *GBA1* variants and the clinical symptoms of different groups. We identified 104 variants, including 8 novel variants, expanding the spectrum of *GBA1* variants. The frequency of *GBA1* variants in patients with PD was 7.46%, higher than that in the control (1.81%) (*P* < 0.001, odds ratio [OR] = 4.38, 95% confidence interval [CI]: 3.26–5.89). Among patients, 176 (4.36%) had severe variants, 34 (0.84%) carried mild variants, three (0.07%) had risk variants, and 88 (2.18%) carried unknown variants. Our study, for the first time, found that p.G241R (*P* = 0.007, OR = 15.3, 95% CI: 1.25–261.1) and p.S310G (*P* = 0.005, OR = 4.86, 95% CI: 1.52–28.04) variants increased the risk of PD. Patients with *GBA1* variants exhibited an earlier onset age and higher risk of probable rapid-eye-movement sleep behavior disorder, olfactory dysfunction, depression, and autonomic dysfunction than patients without *GBA1* variants.

## Introduction

Parkinson’s disease (PD) is a neurodegenerative and progressively disabling disease characterized by bradykinesia, tremor, and muscular rigidity^1^. A multitude of factors can affect the risk, onset, and progression of PD, including aging as well as environmental conditions, and genetic predisposition^1^.

More than twenty genes with different degrees of genetic evidence are mutated in monogenic PD^2,3^. Variants in the *GBA1* gene, encoding the lysosomal enzyme β-glucocerebrosidase (GCase), are common risk factors^4^. *GBA1* variants confer 5–30 folds increased risk of PD, and across different populations, at least 5–20% of patients with PD have *GBA1* variants^5^. More than 300 PD-related variants have been reported, with distinctive patterns observed in different populations^6^.

Studies that explore the correlation between genotype and phenotype have also illuminated how variants of *GBA1* can influence the characteristics of PD^7,8^. Patients harboring *GBA1* variants, when compared to those without, exhibit distinct features, including an earlier age at onset (AAO), more severe motor impairment, higher risk of cognitive decline, depression^7,9,10^, rapid-eye-movement sleep behavior disorder (RBD)^11^, and reduced survival^12^. Furthermore, the clinical attributes resulting from various *GBA1* variants differ. The classification of *GBA1* variants is based on their role in Gaucher’s Disease (GD) or PD: mild variants give rise to GD type I, while severe variants lead to GD type II or III^6^. Presence of heterozygous *GBA1* variants, whether mild or severe, might differentially impact the risk and age at onset of PD^13–15^. Most studies on *GBA1* variants in patients with PD focused on several *GBA1* variants, such as p.L483P, p.N409S, and p.R159W. Although some case-control studies have investigated the coding region of *GBA1* gene in Chinese population, these studies were limited by their relatively small sample sizes^15–17^.

This study aimed to characterize the frequency and distribution of *GBA1* variants in a large cohort of 4034 patients with PD and 2931 healthy participants who are Han Chinese using whole-exome sequencing (WES) and whole-genome sequencing (WGS). All variants were validated by polymerase chain reaction and Sanger sequencing. In addition, we analyzed the relationship between *GBA1* variants and phenotypes by comprehensively assessing the clinical manifestations in patients with PD.

## Results

### Demographic characteristics

This study encompassed a total of 4034 patients and 2931 healthy participants. Among the patients, 1777 (44.1%) individuals were diagnosed with early-onset PD (EOPD, AAO ≤ 50 years); parallelly, 1652 healthy participants matched for age and sex were included. Additionally, there were 2257 (55.9%) participants with late-onset PD (LOPD, AAO > 50 years), accompanied by 1279 age and sex matched control participants. Detailed demographic information for this cohort is presented in Supplementary Table 1. Notably, none of the participants had been previously identified as carriers of pathogenic or likely pathogenic variants associated with PD-causing genes^3,18^.

### Spectrum and frequency of *GBA1* variants

In this study, 104 variants were identified, comprising 84 missense, 6 splicing, 8 frameshift, and 6 stop-gain variants. Among these, 96 variants had been previously reported, while the remaining 8 were novel. Among PD patients, 92 variants were detected, encompassing 72 missense, 6 splicing, 8 frameshift, and 6 stop-gain variants. Notably, 10 variants were shared between PD patients and control, whereas, 12 variants were exclusive to the control group.

**Fig. 1:**
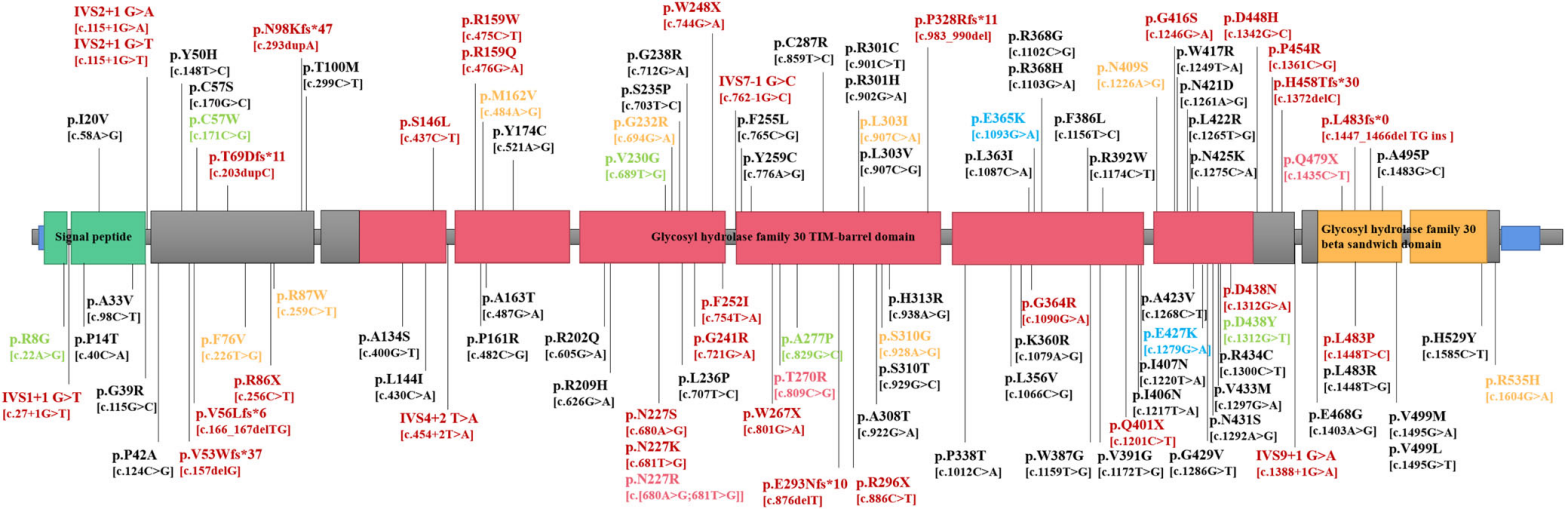
Distribution of *GBA1* variants in patients with PD and control. Schematic drawing of the gene and the protein domains, and diagram of reported and newly discovered *GBA1* variants. Note: Different colors indicate different types of *GBA1* variants. Red indicates reported severe variants, Pink indicates novel severe variants, which means variants are loss of function variants or variants were reported in Gaucher’s patients and were defined severe but were unreported in PD, Yellow indicates mild variants, Blue indicates risk variants, Black indicates reported variants of unknown significance, and green indicates novel missense variants. PD Parkinson’s Disease.

The *GBA1* variants were classified into four different types based on their deduced and observed phenotypic effects on GD or PD. Of all the variants found in PD patients, 34 were classified as severe, 7 as mild, 2 as risk, and 49 as unknown. Of all the variants found in control, four were classified as severe, two as mild, one as risk, and the other 15 were unknown (Fig. 1).

**Fig. 2:**
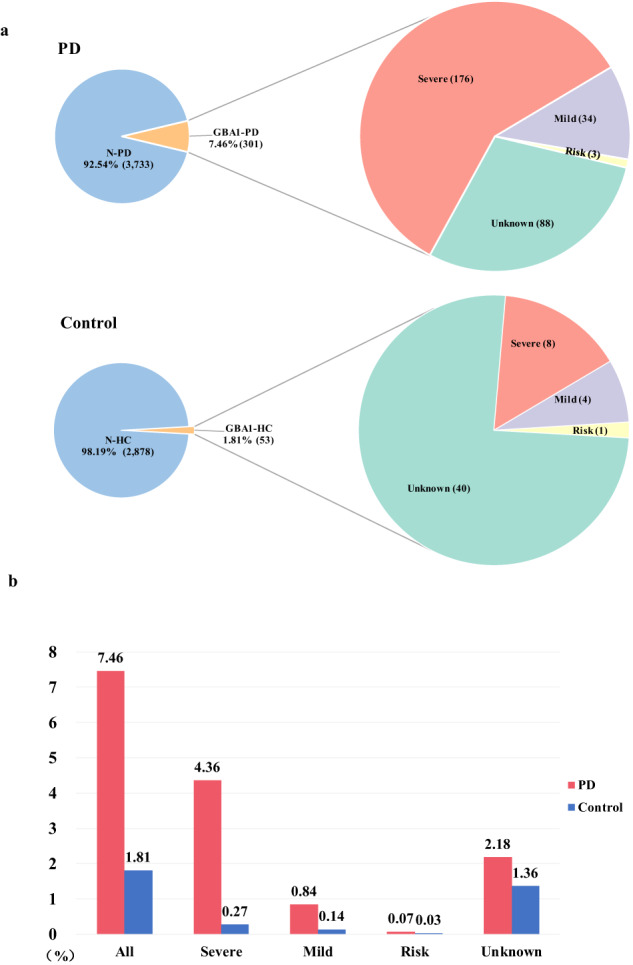
Frequency of *GBA1* variant in patients with PD and control. **a** Number of patients and control with different types of *GBA1* variants. **b** Frequency of different types of *GBA1* variants in patients and control. PD Parkinson’s disease; N-PD patients without *GBA1* variants; GBA1-PD patients with *GBA1* variants; N-HC control without *GBA1* variants; GBA1-HC control with *GBA1* variants; Severe known to cause GD type II or III; Mild known to cause GD type I; Risk variants that are associated with risk for PD but do not cause GD; Unknown reported variants of unknown significance or unreported missense variants; GD Gaucher’s disease.

Furthermore, we observed that, apart from p.L483P and p.R202Q, which were low-frequency variants with a minor allele frequency (MAF) ranging from 0.01 to 0.05, the remaining variants were deemed rare according to the criterion of MAF being <0.01. After comparing the frequency of *GBA1* variants between these two groups, we found that *GBA1* variants detected in patients with PD significantly differed from those observed in control. Among the 4034 patients with PD, 301 (7.46%) carried *GBA1* variants, while 53 (1.81%) out of 2931 control carried *GBA1* variants (*P* < 0.001, odds ratio [OR] = 4.38, 95% confidence interval [CI]: 3.26–5.89) (Fig. 2).

Regarding the types of *GBA1* variants, we identified 176 patients (4.36%) carrying severe variants, a prevalence significantly higher than that among control (0.27%) (*P* < 0.001, OR = 16.67, 95% CI: 8.19–33.91). Mild variants were present in 34 patients (0.84%), while only in four control participants (0.14%) (*P* < 0.001, OR = 6.22, 95% CI: 2.21–17.55). However, the analysis showed no significant difference in the occurrence of risk variants between patients (0.07%) and control (0.03%) (*P* = 0.643, OR = 2.18, 95% CI: 0.23–20.97). For unknown variants, they were identified in 88 patients (2.18%) and 40 control participants (1.36%), demonstrating a statistically significant difference (*P* = 0.016, OR = 1.61, 95% CI: 1.11–2.35) (Fig. 2, Supplementary Table 2).

When selecting variants with at least ten patients to systematically interrogate the association of single-nucleotide variants (SNVs), we observed significant differences for the p.L483P variant detected in PD patients compared to that in control (2.35% vs. 0.14%, *P* < 0.001, OR = 17.65, 95% CI: 6.48–48.05). Furthermore, our study showed that p.G241R and p.S310G variants contribute to an increased risk of PD. Specifically, p.G241R was present in 10 patients (0.25%) but none in control (*P* = 0.007, OR = 15.3, 95% CI: 1.25–261.1). Similarly, p.S310G was found in twenty patients (0.5%) and only three control participants (0.1%) (*P* = 0.005, OR = 4.86, 95% CI: 1.52–28.04). Conversely, the analysis did not reveal significant difference for the second most common variant in our cohort, the p.R202Q variant, with frequencies of 0.55% in patients and 0.72% in control (Table 1, Supplementary Table 3).

**Table 1:** All *GBA1* non-syn synonymous variations identified in this study.

Severity	Chromosomal Position	Exon	cDNA Change	AA Change	Variant Type	Frequency in gnomAD_EAS	CADD	Total		EOPD		LOPD	
								Case	Control	Case	Control	Case	Control
Severe	Chr1:155209424	4	c.437 C > T	p.S146L	Missense	–	29.8:D	1				1	
	Chr1:155208421	5	c.475 C > T	p.R159W	Missense	0	28.6:D	3		1		2	
	Chr1:155208420	5	c.476 G > A	p.R159Q	Missense	–	29.3:D	2		2			
	Chr1:155208006	6	c.[680 A > G;681 T > G]^*^	P.N227R	Missense	–	–	3	1	1	1	2	
	Chr1:155208006	6	c.680 A > G	p.N227S	Missense	0	0.013:T	7		2		5	
	Chr1:155208005	6	c.681 T > G	p.N227K	Missense	0	18.28:T	1		1			
	Chr1:155207965	6	c.721 G > A	p.G241R	Missense	–	24.4:D	10		4		6	
	Chr1:155207932	6	c.754 T > A	p.F252I	Missense	0	23.6:D	2		2			
	Chr1:155207322	7	c.809 C > G^*^	p.T270R	Missense	–	26.5:D	1				1	
	Chr1:155206170	8	c.1090 G > A	p.G364R	Missense	–	18.92:T		1		1		
	Chr1:155205614	9	c.1246 G > A	p.G416S	Missense	0	33:D	2		2			
	Chr1:155205548	9	c.1312 G > A	p.D438N	Missense	–	26.1:D	3				3	
	Chr1:155205518	9	c.1342 G > C	p.D448H	Missense	0.0006	22.7:D	8		5		3	
	Chr1:155205499	9	c.1361 C > G	p.P454R	Missense	–	26.6:D	1		1			
	Chr1:155205043	10	c.1448 T > C	p.L483P	Missense	0.0012	24.8:D	95	4	74	4	21	
	Chr1:155210876	1	c.27+1 G > T		Splicing	–	23.7:D	1				1	
	Chr1:155210420	2	c.115+1 G > A		Splicing	0	25:D	3		2		1	
	Chr1:155210420	2	c.115+1 G > T		Splicing	–	25.1:D	1		1			
	Chr1:155209405	4	c.454+2 T > A		Splicing	–	21.6:D	1		1			
	Chr1:155207370	7	c.762-1 G > C		Splicing	0.0006	23.3:D	8	2	7	2	1	
	Chr1:155205471	9	c.1388+1 G > A		Splicing	–	25.5:D	1		1			
	Chr1:155209728	3	c.256 C > T	p.R86X	Stopgain	–	34:D	1		1			
	Chr1:155207942	6	c.744 G > A	p.W248X	Stopgain	–	38:D	5		3		2	
	Chr1:155207330	7	c.801 G > A	p.W267X	Stopgain	–	37:D	2		2			
	Chr1:155207245	7	c.886 C > T	p.R296X	Stopgain	–	36:D	1		1			
	Chr1:155206059	8	c.1201 C > T	p.Q401X	Stopgain	–	36:D	1		1			
	Chr1:155205056	10	c.1435 C > T^*^	p.Q479X	Stopgain	–	28.7:D	1		1			
	Chr1:155209827	3	c.157delG		Frameshift	–	-	1		1			
	Chr1:155209817	3	c.166_167delTG		Frameshift	–	-	1		1			
	Chr1:155209780	3	c.203dupC		Frameshift	–	-	1		1			
	Chr1:155209690	3	c.293dupA		Frameshift	–	-	1				1	
	Chr1:155207255	7	c.876delT		Frameshift	–	-	1				1	
	Chr1:155207141	7	c.983_990del		Frameshift	–	-	1				1	
	Chr1:155205488	9	c.1372delC		Frameshift	–	-	4		2		2	
	Chr1:155205029	10	c.1447_1466del TG ins		Frameshift	–	-	1		1			
Mild	Chr1:155209758	3	c.226 T > G	p.F76V	Missense	–	12.81:T	1		1			
	Chr1:155209725	3	c.259 C > T	p.R87W	Missense	–	24.8:D		1				1
	Chr1:155208412	5	c.484 A > G	p.M162V	Missense	–	10.47:T	2				2	
	Chr1:155207992	6	c.694 G > A	p.G232R	Missense	–	26.1:D	1				1	
	Chr1:155207224	7	c.907 C > A	p.L303I	Missense	–	24.2:D	8		7		1	
	Chr1:155207203	7	c.928 A > G	p.S310G	Missense	0.0006	14.81:T	20	3	12	3	8	
	Chr1:155205634	9	c.1226 A > G	p.N409S	Missense	0	22.7:D	1				1	
	Chr1:155204793	11	c.1604G>A	p.R535H	Missense	0	15.69:T	2		1		1	
Risk	Chr1:155206167	8	c.1093 G > A	p.E365K	Missense	0	17.33:T	1	1		1	1	
	Chr1:155205581	9	c.1279 G > A	p.E427K	Missense	0	23.2:D	2		1		1	
Unknown	Chr1:155210496	2	c.40 C > A	p.P14T	Missense	–	0.01:T	1				1	
	Chr1:155210478	2	c.58 A > G	p.I20V	Missense	0.0006	0.001:T	1	1		1	1	
	Chr1:155210438	2	c.98 C > T	p.A33V	Missense	0.0006	17.03:T	1		1			
	Chr1:155210421	2	c.115 G > C	p.G39R	Missense	–	24.3:D	3		3			
	Chr1:155209860	3	c.124 C > G	p.P42A	Missense	–	13.28:T		1		1		
	Chr1:155209836	3	c.148 T > C	p.Y50H	Missense	–	4.628:T	1				1	
	Chr1:155209814	3	c.170 G > C	p.C57S	Missense	–	26:D	1				1	
	Chr1:155209685	3	c.299 C > T	p.T100M	Missense	–	21.7:D	1		1			
	Chr1:155209461	4	c.400 G > T	p.A134S	Missense	–	8.905:T	1		1			
	Chr1:155209431	4	c.430 C > A	p.L144I	Missense	–	23.3:D		1		1		
	Chr1:155208414	5	c.482 C > G	p.P161R	Missense	–	24.2:D	1		1			
	Chr1:155208409	5	c.487 G > A	p.A163T	Missense	–	27.6:D	2				2	
	Chr1:155208375	5	c.521 A > G	p.Y174C	Missense	–	23.9:D	1				1	
	Chr1:155208081	6	c.605 G > A	p.R202Q	Missense	0.0025	20.4:D	22	21	8	16	14	5
	Chr1:155208060	6	c.626 G > A	p.R209H	Missense	0	17.53:T		1		1		
	Chr1:155207983	6	c.703 T > C	p.S235P	Missense	0	11.51:T	2		1		1	
	Chr1:155207979	6	c.707 T > C	p.L236P	Missense	–	25.5:D	2		2			
	Chr1:155207974	6	c.712 G > A	p.G238R	Missense	–	28.1:D	1		1			
	Chr1:155207366	7	c.765 C > G	p.F255L	Missense	–	23.8:D	1		1			
	Chr1:155207355	7	c.776 A > G	p.Y259C	Missense	–	25.4:D	1		1			
	Chr1:155207272	7	c.859 T > C	p.C287R	Missense	–	25.1:D	1				1	
	Chr1:155207230	7	c.901 C > T	p.R301C	Missense	–	24.9:D	1		1			
	Chr1:155207229	7	c.902 G > A	p.R301H	Missense	0	17.54:T	1				1	
	Chr1:155207224	7	c.907 C > G	p.L303V	Missense	–	23.4:D	3		3			
	Chr1:155207209	7	c.922 G > A	p.A308T	Missense	–	19.73:T		1		1		
	Chr1:155207202	7	c.929 G > C	p.S310T	Missense	–	17.67:T	1				1	
	Chr1:155207193	7	c.938 A > G	p.H313R	Missense	–	0.002:T		2		2		
	Chr1:155206248	8	c.1012 C > A	p.P338T	Missense	–	14.00:T	1				1	
	Chr1:155206194	8	c.1066 C > G	p.L356V	Missense	–	10.91:T		1		1		
	Chr1:155206181	8	c.1079 A > G	p.K360R	Missense	–	15.02:T	1				1	
	Chr1:155206173	8	c.1087 C > A	p.L363I	Missense	–	23.3:D		1		1		
	Chr1:155206158	8	c.1102 C > G	p.R368G	Missense	–	22.7:D	1				1	
	Chr1:155206157	8	c.1103 G > A	p.R368H	Missense	–	22.6:D	1		1			
	Chr1:155206104	8	c.1156 T > C	p.F386L	Missense	–	16.68:T	1	1		1	1	
	Chr1:155206101	8	c.1159 T > G	p.W387G	Missense	–	17.66:T	1	1			1	1
	Chr1:155206088	8	c.1172 T > G	p.V391G	Missense	–	26.1:D	1		1			
	Chr1:155206086	8	c.1174 C > T	p.R392W	Missense	–	26:D		1		1		
	Chr1:155206043	8	c.1217 T > A	p.I406N	Missense	–	28.8:D	1		1			
	Chr1:155206040	8	c.1220 T > A	p.I407N	Missense	–	28.4:D	1		1			
	Chr1:155205611	9	c.1249 T > A	p.W417R	Missense	–	26.9:D	2		2			
	Chr1:155205599	9	c.1261 A > G	p.N421D	Missense	–	26.2:D	1				1	
	Chr1:155205595	9	c.1265 T > G	p.L422R	Missense	–	28.4:D	5		3		2	
	Chr1:155205592	9	c.1268 C > T	p.A423V	Missense	–	23.6:D		1		1		
	Chr1:155205585	9	c.1275 C > A	p.N425K	Missense	–	27:D	1				1	
	Chr1:155205574	9	c.1286 G > T	p.G429V	Missense	–	33:D	2				2	
	Chr1:155205568	9	c.1292 A > G	p.N431S	Missense	–	23.4:D	4		1		3	
	Chr1:155205563	9	c.1297 G > A	p.V433M	Missense	–	33:D	2				2	
	Chr1:155205560	9	c.1300 C > T	p.R434C	Missense	–	34:D	2		2			
	Chr1:155205088	10	c.1403 A > G	p.E468G	Missense	–	25.2:D		1		1		
	Chr1:155205043	10	c.1448 T > G	p.L483R	Missense	–	24.6:D	2		1		1	
	Chr1:155205008	10	c.1483 G > C	p.A495P	Missense	0	23:D	4	5	3	3	1	2
	Chr1:155204996	10	c.1495 G > A	p.V499M	Missense	0	27.8:D	2		1		1	
	Chr1:155204996	10	c.1495 G > T	p.V499L	Missense	–	20.7:D	1				1	
	Chr1:155204812	11	c.1585 C > T	p.H529Y	Missense	–	23:D	1		1			
	Chr1:155210882	1	c.22 A > G^*^	p.R8G	Missense	–	14.41:T	1		1			
	Chr1:155209813	3	c.171 C > G^*^	p.C57W	Missense	–	26.1:D	1				1	
	Chr1:155207997	6	c.689 T > G^*^	p.V230G	Missense	0	0.206:T	1				1	
	Chr1:155207302	7	c.829 G > C^*^	p.A277P	Missense	–	24.8:D	1				1	
	Chr1:155205548	9	c.1312 G > T^*^	p.D438Y	Missense	–	29.3:D	1		1			
Total								307^a^	53	189^b^	44	118^c^	9

^a^In total cases, five of them carried more than one variant. ^b^In EOPD cases, four of them carried two variants. ^c^ In LOPD cases, one carried three variants. ^*^ First reported in our study. gnomAD_EAS, East Asian population from gnomAD genome.

*PD* Parkinson’s disease, *EOPD* early-onset PD, *LOPD* late-onset PD, *AA* amino acid.

*CADD* Combined Annotation Dependent Depletion, *D* Damaging, *T* Tolerable.

Furthermore, we investigated the frequency of *GBA1* variants in EOPD and LOPD patients. In EOPD patients, 185 (10.41%) carried *GBA1* variants, significantly higher than in LOPD patients, in which only 116 (5.14%) carried *GBA1* variants. A total of 122 (6.87%) EOPD patients and 54 (2.39%) LOPD patients carried severe variants. However, the analysis showed no significant differences for mild, risk, and unknown variants between the two groups. Performing the SNV association, we found that the proportion of EOPD patients with the p.L483P variant was significantly higher than that of LOPD patients. Seventy-four (4.16%) EOPD patients carried p.L483P, while 21 (0.93%) LOPD patients carried p.L483P.

### Genotype-Phenotype

**Fig. 3:**
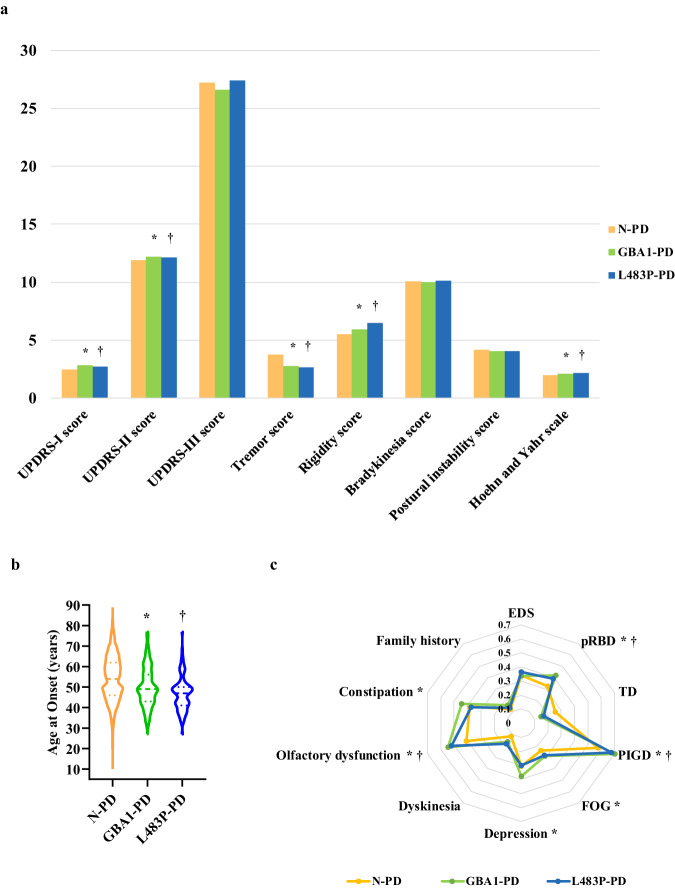
Clinical characteristics of PD with *GBA1* variants. **a** Mean score of motor symptoms in N-PD, GBA1-PD, and L483P-PD. **b** AAO of N-PD, GBA1-PD, and L483P-PD. **c** Frequency (%) of family history and motor and non-motor symptoms in N-PD, GBA1-PD, and L483P-PD. * Significantly different between N-PD and GBA1-PD groups. † Significantly different between N-PD and L483P-PD groups. PD Parkinson’s disease; N-PD patients without *GBA1* variant; GBA1-PD patients with *GBA1* variants; L483P-PD patients with *GBA1* p.L483P variant; AAO age at onset; UPDRS Unified Parkinson’s Disease Rating Scale; EDS excessive daytime sleepiness; pRBD probable rapid-eye-movement sleep behavior disorder; TD tremor-dominant; PIGD postural instability and gait difficulty; FOG Freezing of Gait.

We found that patients with non-synonymous *GBA1* variants had an earlier AAO (mean: 50 years, standard deviation (SD): 9.64 years) compared to non-carriers (mean: 54.15 years, SD: 11.01 years), along with a higher Hoehn and Yahr (H-Y) stage (mean: 2.12, SD: 0.76) in contrast to non-carriers (mean: 1.98, SD: 0.76) (Table 2). Furthermore, the postural instability gait difficulty (PIGD) motor subtype was predominant in both groups, but the proportion of PIGD in patients with *GBA1* variants was higher than that in non-carriers (69.93% vs. 57.36%), indicating increased rigidity and less tremor. In the realm of non-motor symptoms, patients with *GBA1* variants demonstrated lower Hyposmia Rating Scale (HRS) scores related to olfactory function than those without *GBA1* variants (18.19 vs. 19.55), and olfactory loss was more prevalent among patients with *GBA1* variants than non-carriers (54.48% vs. 40.95%). Regarding sleep disturbances, patients with *GBA1* variants exhibited a higher rate of probable rapid-eye-movement sleep behavior disorder (pRBD) than those without *GBA1* variants, while no significant differences were observed in excessive daytime sleepiness (EDS) and overall sleep quality. In addition, patients with non-synonymous *GBA1* variants displayed higher rates of constipation and depression than non-carriers. Regarding motor complications, patients with *GBA1* variants had higher freezing of gait (FOG) rate than non-carriers (Fig. 3, Table 2).

**Table 2:** Comparison of clinical features in N-PD, GBA1-PD and L483P-PD.

Clinical features	N-PD (*N* = 3733)	GBA1-PD (*N* = 301)		L483P-PD (*N* = 95)	
		Values	P1	Values	P2
Age (years)	59.56 ± 10.98	55.30 ± 9.79	**<0.001**	52.32 ± 8.76	**<0.001**
Age at onset (years)	54.15 ± 11.01	50.00 ± 9.64	**<0.001**	46.84 ± 8.38	**<0.001**
Disease duration (years)	5.37 ± 4.39	5.24 ± 4.49	0.404	5.48 ± 5.11	0.12
Sex (male)	1979 (53.01%)	168 (55.81%)	0.528	56 (58.95%)	0.374
Family history	469 (12.56%)	48 (15.95%)	0.144	13 (13.68%)	0.927
UPDRS-I score	2.45 ± 2.04	2.83 ± 2.26	**<0.001**	2.72 ± 2.41	**0.023**
UPDRS-II score	11.89 ± 6.52	12.17 ± 6.69	**0.001**	12.11 ± 6.41	**0.012**
UPDRS-III score	27.22 ± 14.86	26.60 ± 14.39	0.175	27.41 ± 14.71	0.101
Tremor score	3.76 ± 3.64	2.75 ± 3.10	**<0.001**	2.65 ± 3.06	**0.008**
Rigidity score	5.49 ± 4.15	5.93 ± 4.37	**0.012**	6.49 ± 4.40	**0.026**
Bradykinesia score	10.07 ± 6.36	9.99 ± 5.97	0.186	10.13 ± 6.09	0.171
Postural instability score	4.16 ± 3.03	4.07 ± 2.91	0.226	4.06 ± 2.95	0.203
Hoehn and Yahr Scale	1.98 ± 0.76	2.12 ± 0.76	**<0.001**	2.16 ± 0.75	**<0.001**
Hoehn and Yahr stage			**0.001**		**<0.001**
1–1.5	1161 (31.09%)	78 (25.91%)		22 (23.16%)	
2–2.5	1602 (42.92%)	148 (49.17%)		47 (49.47%)	
3–5	970 (25.99%)	75 (24.92%)		26 (27.37%)	
Motor subtype					
TD	950 (25.44%)	45 (14.86%)		16 (16.84%)	
Indeterminate	642 (17.20%)	46 (15.20%)	0.071	15 (15.79%)	0.344
PIGD	2141 (57.36%)	210 (69.93%)	**<0.001**	64 (67.37%)	**0.003**
MMSE score	26.35 ± 3.90	26.19 ± 4.01	**0.004**	26.27 ± 4.51	**0.03**
PDSS score	115.53 ± 28.06	115.93 ± 26.16	0.354	116.14 ± 27.54	0.495
ESS score	7.78 ± 6.30	7.68 ± 6.37	0.27	7.62 ± 6.66	0.368
EDS	1109 (34.22%)	87 (33.72%)	0.244	28 (36.36%)	0.119
RBDQ total score	16.11 ± 16.57	20.50 ± 18.99	**<0.001**	19.96 ± 19.57	**0.002**
pRBD	1163 (32.48%)	117 (42.31%)	**<0.001**	30 (38.96%)	**0.016**
HAMD score	5.81 ± 5.51	6.37 ± 5.69	**0.048**	6.13 ± 6.59	0.432
Depression	980 (30.39%)	97 (37.89%)	**0.006**	23 (30.26%)	0.93
HRS score	19.55 ± 6.54	18.19 ± 6.44	**<0.001**	18.58 ± 6.25	**0.029**
Olfactory dysfunction	1330 (40.95%)	146 (54.48%)	**<0.001**	44 (51.76%)	**0.002**
PDQ39 score	29.50 ± 25.69	30.28 ± 25.59	0.068	26.23 ± 23.68	0.791
Constipation	1207 (38.32%)	111 (44.22%)	**<0.001**	30 (37.04%)	0.096
Dyskinesia	433 (11.59%)	49 (16.04%)	0.053	17 (18.09%)	0.324
Freezing gait	903 (24.18%)	86 (28.67%)	**0.048**	27 (28.42%)	0.119
LEDD (mg)	402.59 ± 285.39	439.48 ± 230.76	**0.036**	476.08 ± 252.23	**0.027**

Values are expressed as mean ± standard deviation, or number (%).

*PD* Parkinson’s disease, *N-PD* patients without *GBA1* variants, *GBA1-PD* patients with *GBA1* variants, *L483P-PD* patients with p.L483P variant. *UPDRS* Unified Parkinson’s disease Rating Scale, *TD* tremor-dominant, *PIGD* postural instability and gait difficulty, *MMSE* Mini-Mental State Examination, *PDSS* Parkinson’s Disease Sleep Scale, *ESS* Epworth Sleepiness Scale, *EDS* excessive daytime sleepiness, *RBDQ* Rapid-eye-movement Sleep Behavior Disorder Questionnaire, *pRBD* probable rapid-eye-movement sleep behavior disorder, *HAMD* Hamilton Depression Scale, *HRS* Hyposmia Rating Scale, *PDQ-39* Parkinson Disease Quality of Life Questionnaire-39 item version, *LEDD* levodopa equivalent daily dose. The scores of UPDRS items 20 and 21 added up to the tremor score. The score for item 22 was the rigidity score. The scores for items 23 to 26 added up to the bradykinesia score. The scores for items 27–30 added up to the postural instability score. Disease motor subtype was classified as tremor-dominant (TD) phenotype when the ratio of tremor score and postural instability and gait difficulty (PIGD) score was no less than 1.5, while patients with a ratio of no more than 1.0 were defined to PIGD phenotype and rest of patients belonged to the indeterminate phenotype.

Since the most important variant we found was p.L483P, we specifically analyzed the clinical characteristics of patients with p.L483P. Compared with non-carriers, cases with p.L483P had an earlier AAO and a higher H-Y stage. Compared with non-carriers, olfactory loss and pRBD were more prevalent in those with p.L483P (Fig. 3, Table 2).

Additionally, we analyzed the clinical characteristics of both EOPD and LOPD cases with *GBA1* variants. When comparing EOPD cases with and without *GBA1* variants, we found no significant differences between the two groups in age and AAO. However, patients with *GBA1* variants displayed a higher H-Y stage. Moreover, compared to non-carriers, patients with *GBA1* variants exhibited a higher prevalence of olfactory loss, pRBD, and constipation. While when comparing LOPD cases with and without *GBA1* variants, those with *GBA1* variants exhibited an earlier AAO. Furthermore, *GBA1* variants carriers within the LOPD group displayed a higher occurrence of olfactory loss, depression, pRBD and constipation (Supplementary Table 4).

Finally, we compared the clinical characteristics of different types of *GBA1* variants including Severe-PD and Mild-PD. Notably, Severe-PD cases displayed an earlier AAO and higher levodopa equivalent daily dose (LEDD) compared to Mild-PD cases. However, we did not identify significant differences in other clinical characteristics (Supplementary Table 5).

## Discussion

This study represents the largest endeavor to comprehensively analyze *GBA1* coding variants within a Chinese cohort of patients with PD and control. This investigation identified 104 variants, including 8 novel variants, thereby expanding the spectrum of *GBA1* variants. Notably, the frequency of *GBA1* variants among patients with PD was 7.46%, significantly higher than that in control (1.81%). This observed frequency among patients with PD was within the reported range (5.4–10.72%) in other Chinese studies to date^15–17,19,20^. Moreover, when categorizing these variants according to their deduced and observed phenotypic impact on GD or PD, a significant proportion of patients with PD carried the *GBA1* variants associated with phenotypic effects.

Although globally, four missense variants-p.E365K, p.T408M, p.N409S, and p.L483P-account for >80% of PD alleles^21^, we found no patients with p.T408M, and only two patients had p.N490S and p.E365K, respectively. Moreover, these three variants are very rare among East Asians and are present in up to only 0.2% of South Asians based on the gnomAD database. The p.L483P variant was prominent among our Chinese patients with PD, which, together with p.R202Q, p.S310G, and p.G241R, accounted for half of the *GBA1* cases in this group. Our study showed that p.L483P, p.S310G, and p.G241R increased the risk of PD. The p.L483P variant has also been reported to be the most common *GBA1* variant in other Asian and Hispanic populations^16,20^. While among Ashkenazi Jews, the *GBA1* variants in PD patients were mainly p.N409S^22^. The difference in mutation frequency indicates that it may be affected by factors such as environment, region, ethnicity, etc. Interestingly, our study firstly found p.S310G and p.G241R may be relatively specific variants for increasing the risk of PD in Chinese population.

In terms of clinical characteristics, *GBA1* variant carriers had an average of 4 years earlier in age at onset than non-carriers in our study, consistent with previous studies that found PD patients with *GBA1* variants were younger and had 1–11 years earlier in age at onset than PD patients without *GBA1* variants^23–25^. In addition, we found that the tremor score of PD patients with *GBA1* variants was relatively lower and the PIGD motor subtype was more common in PD patients with *GBA1* variants than in patients without *GBA1* variants, indicating that PD patients with *GBA1* variants may belong to the non-tremor-dominant phenotype of PD. This finding is consistent with findings from a recent study that patients with *GBA1* variants were more likely to present with the PIGD phenotype compared with non-carriers^26^. In addition, one study found that patients with PD who carried *GBA1* variants displayed a faster decline in PIGD scores but not tremor scores^27^.

Previous studies have reported that, PD patients carrying *GBA1* variants showed worse cognitive function than PD patients who did not carry *GBA1* variants^26^. Some studies also showed that there was no significant difference in cognitive function between PD patients with *GBA1* variants and PD patients without *GBA1* variants in the early stages of the disease. As the disease progresses, PD patients with *GBA1* variants progress to dementia faster^28^. In our study, there was a statistical difference in cognitive function between PD patients with *GBA1* variants and PD patients without *GBA1* variants, while the difference of Mini-Mental State Examination (MMSE) scores was marginal. This could be attributed to the average course duration of our patients, which was only about 5 years, shorter than those reported with differences in cognitive function. Whether the decline in cognitive function is faster in PD patients with *GBA1* variants requires further conclusions through prospective studies.

Our study also analyzed the relationship between *GBA1* variants and phenotypes by comprehensively assessing the clinical manifestations in patients with PD. Consistent with previous reports, we found that PD patients with *GBA1* variants were likelier to develop olfactory dysfunction. In addition, we found that depression was likelier to occur in PD patients with *GBA1* variants. Previous studies have reported that PD patients with *GBA1* variants have olfactory disturbances and depression at the same time^29^ This observation could be attributed to the olfactory pathway affecting the serotonin circuit in the body, affecting the hippocampus, amygdala, and other emotional centers^30^. Regarding sleep, we found that PD patients with *GBA1* variants were likelier to develop pRBD, which is consistent with the findings of previous studies of patients carrying *GBA1* variants with a significantly higher risk of RBD^7,11^. However, no significant difference was found between excessive daytime sleepiness and overall sleep quality. Regarding autonomic function, PD patients with *GBA1* variants were more prone to constipation. Lastly, we found that PD patients with *GBA1* variants were likelier to have freezing of gait, indicating that *GBA1* variants may be an important risk factor affecting the occurrence of freezing of gait in PD patients.

In addition, we also explored the role of different types of *GBA1* variants including Severe-PD and Mild-PD in PD risk and the clinical characteristics. We found that patients with severe variants had a higher OR (16.67) compared to patients with mild *GBA1* variants (6.22), which is consistent with the findings of a large meta-analysis demonstrating that patients with mild variants have a lower OR (2.2) compared to patients with severe variants (10.3)^13^. Furthermore, we found that patients with severe variants had an earlier average AAO and a higher LEDD relative to those with mild *GBA1* variants, which is in agreement with previously reported studies^7,13,14^. Previous studies have reported that Severe-PD, compared to Mild-PD, presented with worse motor and non-motor manifestations of PD, including more severe cognitive dysfunction, hyposmia, depression, and a higher frequency RBD^7^. However, our study did not yield significant differences between the two groups in relation to these clinical characteristics. Although the findings did not achieve statistical significance, it’s worth noting that the frequency of pRBD was conspicuously higher in Severe-PD compared to Mild-PD. The potential necessity for larger cohorts to effectively detect such differences is a consideration.

Nonetheless, our study has several limitations. Firstly, we did not carry out functional verification of novel variants. Additionally, we did not measure GCase activity, which has been linked to PD. Moreover, our sequencing approach was not uniform. Due to limited funding and technology constraints, we initially focused on genetic information of early-onset PD (AAO <50 years old) and PD with a family history, sequenced via WES. Subsequently, in the second stage of the project, we directed our attention to the genetic information of idiopathic PD patients with late-onset (AAO > 50) and sequenced them using WGS, driven by advancements in sequencing technology. To further our understanding, we have initiated the Chinese Parkinson’s Disease with *GBA1* Variants Registry (CPD-GBAR) study, a multicenter, nationwide PD cohort study (the clinicaltrials.gov identifier is NCT03523065) based on the PD-MDCNC. This study aims to explore disease progression, genetic modifying factors, and more in patients with *GBA1* variants.

In conclusion, our study not only expanded the spectrum of *GBA1* variants by identifying 8 novel *GBA1* variants but also underscored the relatively high prevalence of PD patients carrying *GBA1* variants within the Chinese population. Notably, the p.L483P variant emerged as the most frequent risk factor. Additionally, for the first time, we revealed that p.S310G and p.G241R variants contributed to an increased risk of PD. Furthermore, our findings offered insights into the clinical spectrum of *GBA1* variation in Chinese population and furnish valuable *GBA1* genotype-phenotype observations. Patients with *GBA1* variants, compared to those without *GBA1* variants, exhibited an earlier age at onset, and higher risk of pRBD, olfactory dysfunction, depression, and autonomic dysfunction.

## Methods

### Participants

Participants were recruited between October 2006 and August 2021 at the Xiangya Hospital Central South University, as well as other sites affiliated with the Parkinson’s Disease and Movement Disorders Multicenter Database and Collaborative Network in China (PD-MDCNC, http://pd-mdcnc.com). All the PD patients received diagnoses from experienced neurologists, adhering to either the UK Brain Bank Clinical Diagnostic Criteria for PD or the 2015 International Parkinson and Movement Disorder Society Clinical Diagnostic Criteria for PD. Neurological disease-free control participants consisted of community volunteers and spouses of the patients. Each participant provided informed consent prior to their involvement, and this study was approved by the Ethics Committee of Xiangya Hospital of Central South University. Notably, all participants were self-reported Chinese Han ethnic.

### Clinical assessment

Demographic and clinical data were collected, including age, sex, family history, disease duration, and motor and non-motor manifestations. The Unified Parkinson’s Disease Rating Scale (UPDRS)^31^ and the Hoehn and Yahr (H-Y) scale^32^ were used to evaluate motor severity. Patients were assessed during “OFF” medication conditions. UPDRS items for TD and PIGD designations were used to calculate mean TD and PIGD scores. Following the original classification methods, the ratio of the mean UPDRS tremor scores (8 items) to the mean UPDRS PIGD scores (5 items) was used to define TD subtype (ratio ≥ 1.5), PIGD subtype (ratio ≤ 1), and indeterminate subtype (ratio > 1.0 and <1.5)^33^. The 17-item Hamilton Depression Rating Scale (HAMD-17)^34^ was used to evaluate depression, and a score of it <7 points suggests no depression. Sleep status was evaluated using the REM Sleep Behavior Disorder Questionnaire-Hong Kong (RBDQ-HK)^35^, Epworth Sleepiness Scale (ESS)^36^ and Parkinson’s Disease Sleep Scale (PDSS)^37^. A score of ESS ≥ 10 points represents excessive daytime sleepiness, while a factor 2 score of RBDQ-HK ≥ 7 or a total score ≥18 classifies pRBD. The olfactory function was evaluated using the Hyposmia Rating Scale (HRS)^38,39^, and a score of it ≤22 indicates hyposmia. The Mini-Mental State Examination (MMSE)^40^ was used to evaluate cognitive function. The Functional Constipation Diagnostic Criteria Rome III (ROME III)^41^ and the Scale for Outcomes in Parkinson’s disease for Autonomic Symptoms (SCOPA-AUT)^42^ were used to evaluate constipation status. Dyskinesia and freezing gait were evaluated using the Dyskinesia Screening Scale^43^ and the Freezing Gait (FOG) Scale^44^, respectively. The 39-item Parkinson’s disease questionnaire (PDQ-39)^45^ was used to evaluate quality of life^46^.

### Genotyping

Genomic DNA was extracted from peripheral blood leukocytes following standard procedures. Variants within PD patients with age at onset (AAO) of 50 years or younger and those with a family history of PD and control participants without neurological disease were identified through WES. Meanwhile, variants within sporadic late-onset PD cases (AAO > 50) and matched healthy control were identified through WGS. The data generation and quality control procedures for the WES and WGS data have been detailed previously^47^. Briefly, the sequencing data were first processed using a bioinformatics pipeline for WES and WGS sequencing data (BWA-GATK-ANNOVAR)^48^, and subsequently, the PLINK software was used to perform a series of quality control procedures for individuals and variant^49^. Similar to the quality control standards used in our earlier study^47^, the high-quality variants were extracted: allele depth (AD) ≥ 5, total depth (DP) ≥ 10, genotype quality (GQ) ≥ 20, and missingness rate <5% for variants from the WES cohort, whereas AD ≥ 2, DP ≥ 5, GQ ≥ 15 for SNPs, GQ ≥ 30 for indels, and missingness rate <5% for variants from the WGS cohort. High-quality variants are located in the *GBA1* transcript region and 2 bp of the boundary region between exons and introns, relative to transcript NM_000157 (chr1: 155204243–155211040; hg19). Of note, patients with pathogenic/likely pathogenic variants of PD-causing genes (*SNCA*, *PRKN*, *UCHL1*, *PINK1*, *DJ1*, *LRRK2*, *ATP13A2*, *GIGYF2*, *HTRA2*, *PLA2G6*, *FBXO7*, *VPS35*, *EIF4G1*, *DNAJC6*, *SYNJ1*, *TMEM230*, *CHCHD2*, *VPS13C*, *RIC3*, *DNAJC13*, *LRP10*, *RAB39B*, *POLG*, *DAGLB*) from the WES cohort were excluded from this study, as described in our previous study^3,18^.

### Variant verification

We have performed validation experiments of *GBA1* variants using a Sanger sequencing method. For the variants, primer design was performed using the Primer 3.0 online primer design database. Given the presence of a pseudogene with high homology to *GBA1* gene, we deliberately selected fragments for primer design located exclusively within the *GBA1* gene, effectively avoiding any overlap with the pseudogene. Related primers were shown in Supplementary Table 6 and the TaKaRa Premix Ex Taq™ DNA Polymerase Hot Start Version (Takara Bio RR030A) was used to amplify different exons of *GBA1* gene. The cycling conditions for amplification were as follows: initial denaturation at 95 °C for 5 min, 30 cycles of denaturation at 95 °C for 30 s, annealing at 55 °C for 30 s, and extension at 72 °C for 1 min. Lastly, samples were held at 4 °C.

Specifically, three exons (exon 1, exon 3 and exon 5) were amplified using previously described primers^50^. *GBA1* was amplified in a large fragment: a 2972 bp fragment encompassing exons 1–5 using previously described primers and a unique 64 °C to 54 °C touch-down PCR program. PCR products were sequenced with internal primers, adjacent to coding exons and exon-intron boundaries. Related primers were shown in Supplementary Table 6.

### Classification of *GBA1* Variants

The *GBA1* variants were classified into four different types based on their deduced and observed phenotypic effects on GD or PD: severe variants (known to cause GD type II or III), mild variants (known to cause GD type I), risk variants (variants that are associated with risk for PD but do not cause GD), and unknown variants (reported variants of unknown significance or unreported missense variants^6^).

### Statistical analysis

SPSS version 26.0 (IBM SPSS Statistics for Windows, Version 26.0. Armonk, NY: IBM Corp.) was used to analyze the data. Analysis was adjusted for age of onset, disease duration, sex and LEDD, and multiple comparison using Bonferroni corrections (*p* < 0.05). Significance was determined for all analyses if alpha was <0.05 (corrected). Chi-square tests and Fisher exact tests were used to analyze the influence of *GBA1* variants on the onset of PD. Because no control carried the p.G241R variant, we used the Haldane-Anscombe correction to calculate OR. Briefly, we added “0.5” to numbers in each cell of the 2×2 Table and then calculated the OR over these adjusted cell counts. Linear regression was used to compare demographic data with covariate adjustments. The connection between genetic status and clinical manifestations was evaluated through linear regressions, in which continuous scores correlated with genetic status. This analysis was adjusted for variables including age of onset, disease duration, sex, and LEDD. Meanwhile, the correlations between symptom status (H-Y stage, motor complications or non-motor symptoms) and genetic status were analyzed using logistic regression adjusting for age of onset, disease duration, sex and LEDD. Furthermore, an analysis of motor subtypes was conducted using multinomial logistic regression, using the tremor-dominant group as the reference group.

### Supplementary information

Supplementary tables

## Data Availability

The summary data of *GBA1* variants can be accessed after an approved application to the Open Archive for Miscellaneous Data (OMIX) of National Genomic Data Center (NGDC). The accession code is OMIX004514. The clinical data used in this study are owned by PD-MDCNC (http://pd-mdcnc.com). There are no current sharing agreements, and data were held under a data use contract with PD-MDCNC.

## Code availability

The related codes and scripts used in this study don’t present restrictions and are available upon request for information that is made and that can be provided by the corresponding author.
