# Supplementary material for: Mutational spectrum and clinical features of GBA1 variants in a Chinese cohort with Parkinson’s disease
Source: NPJ Parkinsons Dis. 2023 Sep 1;9:129. doi: 10.1038/s41531-023-00571-4 (PMC10474275; doi:10.1038/s41531-023-00571-4)
Supplement: Supplementary file 1 — Supplementary tables [file 41531_2023_571_MOESM1_ESM.pdf]

Supplementary Table 1. Basic demographic characteristics of included subjects

Group	Group 1		Group 2	
	EOPD	Control	LOPD	Control
Number	1777	1652	2257	1279
Sequence Method	WES		WGS (except FPD who use WES)	
Age	49.81±7.18	62.03 ± 12.59	66.62± 7.04	62.32 ± 7.10
Age at Onset	43.81±5.75	-	61.74± 6.87	-
Sex (male/female)	987/789	795/857	1160/1097	613/666

WES, whole-exome sequencing; WGS,whole-genome sequencing.

Supplementary Table 2. Analysis of different *GBA1* variant types in cases and health controls.

**Variants groups**	**Case**	**Control**	**OR**	**95%CI**	**P**
All	301	53	4.38	3.26-5.89	**<0.001**
Severe	176	8	16.67	8.19-33.91	**<0.001**
Mild	34	4	6.22	2.21-17.55	**<0.001**
Risk	3	1	2.18	0.23-20.97	0.643
Unknown	88	40	1.61	1.11-2.35	**0.016**

Severe, known to cause GD type II or III; Mild, known to cause GD type I; Risk, variants that are associated with risk for PD but do not cause GD; Unknown, reported variants of unknown significance or unreported missense variants; GD, Gaucher disease; OR, odds ratio; CI, confidence interval; Chi-square test and Fisher exact test were used to analyze.

Supplementary Table 3. Analysis of single *GBA1* variant in cases and health controls.

**Variant**	**Case**	**Control**	**OR**	**95%CI**	**P**
p.L483P	95	4	17.65	6.48-48.05	**<0.001**
p.S310G	20	3	4.86	1.52-28.04	**0.005**
p.G241R	10	0	15.3	1.25-261.1	**0.007**
p.R202Q	22	21	0.76	0.417-1.384	0.439

OR, odds ratio; CI, confidence interval.Variants with the number of cases not less than ten were selected to analysis. Chi-square test and Fisher exact test were used to analyze. Haldane-Anscombe correction was used to calculate OR of p.G241R.

Supplementary Table 4. Demographic and clinical features in GBA1-EOPD vs. N-EOPD and GBA1-LOPD vs. N-LOPD

Clinical features	N-EOPD (N=1,592)	GBA1-EOPD (N=185)		L483P-EOPD (N=74)		N-LOPD (N=2,141)	GBA1-LOPD (N=116)		L483P-LOPD (N=21)	
		Values	p	Values	p		Values	p	Values	p
Age (years)	49.86±7.26	49.38±6.40	0.964	49.19±6.58	0.685	66.72±7.06	64.70±6.22	**0.002**	63.33±6.22	**0.027**
Age at onset (years)	43.81±5.82	43.82±5.15	0.957	43.51±5.35	0.689	61.84±6.88	59.86±6.34	**0.002**	58.57±6.30	**0.027**
Disease duration (years)	6.03±5.15	5.52±5.09	0.321	5.68±5.56	0.871	4.88±3.66	4.80±3.31	0.475	4.79±3.03	0.565
Sex (male)	873 (54.84%)	114 (61.62%)	0.77	48 (64.86%)	0.107	1,106 (51.66%)	54 (46.55%)	0.355	8 (38.10%)	0.261
Family history	195 (12.25%)	27 (14.59%)	0.378	9 (12.16%)	0.913	274 (12.80%)	21 (18.10%)	0.145	4 (19.05%)	0.487
UPDRS-I score	2.27±1.99	2.76±2.30	**0.001**	2.58±2.26	0.063	2.59±2.08	2.94±2.21	**0.026**	3.19±2.87	0.113
UPDRS-II score	11.32±6.65	11.68±6.84	**0.023**	11.69±6.20	0.051	12.31±6.39	12.94±6.41	**0.022**	13.57±7.05	0.107
UPDRS-III score	26.22±15.63	25.56±14.54	0.809	27.11±14.04	0.19	27.96±14.22	28.23±14.06	0.06	28.48±17.19	0.367
Tremor score	3.89±3.77	2.80±2.99	**0.004**	2.72±2.84	**0.014**	3.67±3.53	2.67±3.29	**0.044**	2.43±3.83	0.321
Rigidity score	5.38±4.15	5.87±4.52	0.225	6.59±4.45	**0.036**	5.57±4.15	6.03±4.15	**0.012**	6.14±4.29	0.425
Bradykinesia score	9.76±6.59	9.62±6.11	0.881	9.88±5.89	0.41	10.29±6.17	10.58±5.72	0.051	11.00±6.83	0.228
Postural instability score	3.74±3.10	3.60±2.75	0.347	3.92±2.82	**0.023**	4.48±2.94	4.79±3.01	**0.023**	4.57±3.39	0.394
Hoehn and Yahr Scale	1.89±0.77	2.13±0.75	**<0.001**	2.26±0.74	**<0.001**	2.04±0.75	2.12±0.77	**0.027**	1.81±0.70	0.346
Hoehn and Yahr stage			**<0.001**		**<0.001**			0.262		0.289
1-1.5	594 (37.32%)	52 (28.11%)		14 (18.92%)		567 (26.48%)	26 (22.42%)		8 (38.10%)	
2-2.5	648 (40.70%)	89 (48.11%)		37 (50.00%)		954 (44.56%)	59 (50.86%)		10 (47.62%)	
3-5	350 (21.98%)	44 (23.78%)		23 (31.08%)		620 (28.96%)	31 (26.72%)		3 (14.28%)	
Motor subtype										
TD	447 (28.06%)	32 (17.22%)		13 (17.57%)		503 (23.49%)	13 (11.21%)		3 (14.29%)	
Indeterminate	288 (18.11%)	30 (16.11%)	0.137	10 (13.51%)	0.512	353 (16.49%)	16 (13.79%)	0.321	5 (23.81%)	0.546
PIGD	857 (53.83%)	123 (66.67%)	**<0.001**	51 (68.92%)	**0.003**	1,285 (60.02%)	87 (75.00%)	**0.022**	13 (61.90%)	0.657

MMSE score	27.44±2.95	27.18±3.11	0.102	27.16±3.08	0.268	25.67±4.26	24.87±4.66	**0.012**	23.81±6.62	**0.022**
PDSS score	118.59±28.41	118.71±26.77	0.678	118.88±27.40	0.853	113.72±27.59	112.22±24.97	0.434	108.50±27.17	0.345
ESS score	6.66±5.87	7.47±6.50	0.063	7.96±6.87	0.074	8.45±6.46	7.95±6.23	0.769	6.71±6.16	0.412
EDS	320 (26.62%)	45 (31.03%)	0.144	20 (35.71%)	0.086	789 (38.68%)	42 (37.17%)	0.86	8 (38.10%)	0.724
RBDQ total score	12.87±15.21	18.86±19.73	**<0.001**	19.07±20.37	**0.001**	17.98±17.04	22.88±17.78	**0.001**	23.37±17.34	0.146
pRBD	318 (24.73%)	53 (35.57%)	**0.001**	18 (32.14%)	0.114	845 (37.04%)	64 (51.35%)	**0.001**	12 (57.14%)	**0.036**
HAMD score	5.65±5.57	6.23±6.03	0.123	6.09±6.72	0.419	5.91±5.48	6.56±5.21	0.24	6.25±6.35	0.873
Depression	365 (30.22%)	51 (34.69%)	0.17	17 (30.36%)	0.854	615 (30.49%)	46 (42.20%)	**0.012**	6 (30.00%)	0.874
HRS score	20.46±6.04	18.79±6.39	**0.001**	18.57±6.00	**0.017**	19.02±6.74	17.42±6.44	**0.005**	18.62±7.03	0.613
Olfactory dysfunction	403 (33.53%)	75 (48.39%)	**<0.001**	33 (51.56%)	**0.02**	927 (45.31%)	71 (62.83%)	**<0.001**	11 (52.38%)	0.396
PDQ39 score	27.98±25.08	29.05±26.30	0.194	26.54±24.08	0.993	30.39±26.01	31.87±24.66	0.237	25.43±23.15	0.532
Constipation	303 (25.85%)	56 (36.60%)	**<0.001**	21 (34.43%)	**0.042**	904 (45.70%)	55 (56.12%)	**0.009**	9 (45.00%)	0.731
Dyskinesia	241 (15.14%)	33 (17.83%)	0.174	16 (21.62%)	0.108	191 (8.92%)	15 (12.93%)	0.272	1 (4.76%)	0.212
Freezing gait	410 (25.75%)	50 (27.02%)	0.434	22 (29.73%)	0.076	492 (22.98%)	36 (31.03%)	0.11	5 (23.81%)	0.927
LEDD (mg)	428.05±305.19	429.92±219.49	0.476	446.87±218.21	0.369	388.97±273.34	453.76±247.17	**0.035**	520.88±308.31	**0.019**

Values are expressed as mean ± standard deviation, or number (%).

PD, Parkinson's disease; EOPD, early-onset PD; LOPD, late-onset PD; N-EOPD, EOPD patients without *GBA1* variants; GBA1-EOPD, EOPD patients with *GBA1* variants; L483P-EOPD, EOPD patients with p.L483P variant; N-LOPD, LOPD patients without *GBA1* variants; GBA1-LOPD, LOPD patients with *GBA1* variants; L483P-LOPD, LOPD patients with p.L483P variant. UPDRS, Unified Parkinson's disease Rating Scale; TD, tremor-dominant; PIGD, postural instability and gait difficulty; MMSE, Mini-Mental State Examination; PDSS, Parkinson's Disease Sleep Scale; ESS, Epworth Sleepiness Scale; EDS, excessive daytime sleepiness; RBDQ, Rapid-eye-movement Sleep Behavior Disorder Questionnaire; pRBD, probable rapid-eye-movement sleep behavior disorder; HAMD, Hamilton Depression Scale; HRS, Hyposmia Rating Scale; PDQ-39, Parkinson Disease Quality of Life Questionnaire-39 item version, LEDD, levodopa equivalent daily dose. The scores of UPDRS items 20 and 21 added up to the tremor score. The score for item 22 was the rigidity score. The scores for items 23 to 26 added up to the bradykinesia score. The scores for items 27 to 30 added up to the postural instability score. Disease motor subtype was classified as tremor-dominant (TD) phenotype when the ratio of tremor score and postural instability and gait difficulty (PIGD) score was no less than 1.5, while patients with a ratio of no more than 1.0 were defined to PIGD phenotype and rest of patients belonged to the indeterminate phenotype.

Supplementary Table 5. Comparison of clinical features between patients with different *GBA1* variant types

Clinical features	Severe-PD (N=176)	Mild-PD (N=34)	P
Age (years)	53.74±9.21	57.77±9.85	**0.044**
Age at onset (years)	48.41±8.68	51.32±10.02	**0.046**
Disease duration (years)	5.33±4.68	6.43±4.96	0.12
Sex (male)	92 (52.27%)	18 (52.94%)	0.71
Family history	31 (17.61%)	3 (8.82%)	0.21
UPDRS-I score	2.80±2.38	2.91±2.01	0.91
UPDRS-II score	12.20±6.59	13.38±8.65	0.97
UPDRS-III score	27.55±14.58	28.12±17.62	0.58
Tremor score	2.87±3.29	2.59±3.58	0.59
Rigidity score	6.47±4.51	5.88±4.60	0.23
Bradykinesia score	10.25±6.05	11.24±6.94	0.82
Postural instability score	4.02±2.86	4.74±3.93	0.62
Hoehn and Yahr Scale	2.18±0.76	2.31±0.90	0.95
Hoehn and Yahr stage			0.98
1-1.5	73 (41.48%)	13 (38.24%)	
2-2.5	76 (43.18%)	13 (38.24%)	
3-5	27 (15.34%)	8 (23.52%)	
Motor subtype			
TD	28 (15.91%)	2 (5.88%)	
Indeterminate	29 (16.48%)	6 (17.65%)	
PIGD	119 (67.61)%	26 (76.47%)	
MMSE score	26.04±4.28	26.47±3.35	0.15
PDSS score	114.22±25.08	116.46±26.64	0.34
ESS score	7.54±6.61	7.69±6.76	0.94
EDS	53 (35.33%)	8 (30.77%)	0.45
RBDQ total score	21.66±19.93	18.52±17.93	0.20
pRBD	69 (44.66%)	11 (37.93%)	0.20
HAMD score	6.69±6.06	6.93±5.09	0.99
Depression	60 (40.54%)	13 (46.43%)	0.67
HRS score	18.28±6.39	18.64±6.31	0.65
Olfactory dysfunction	83 (52.53%)	16 (57.14%)	0.78
PDQ39 score	30.72±26.28	33.32±26.67	0.90
Constipation	64 (43.54%)	9 (37.5%)	0.23
Dyskinesia	34 (19.32%)	4 (11.76%)	0.20
Freezing gait	52 (29.55%)	11 (32.35%)	0.93
LEDD (mg)	462.22±239.82	359.17±186.23	**0.017**

Values are expressed as mean ± standard deviation, or number (%).

PD, Parkinson's disease; Severe-PD, patients with severe variants;Mild-PD, patients with mild variants; UPDRS, Unified Parkinson's disease Rating Scale; TD, tremor-dominant; PIGD, postural instability and gait difficulty;

MMSE, Mini-Mental State Examination; PDSS, Parkinson's Disease Sleep Scale; ESS, Epworth Sleepiness Scale; EDS, excessive daytime sleepiness; RBDQ, Rapid-eye-movement Sleep Behavior Disorder Questionnaire; pRBD, probable rapid-eye-movement sleep behavior disorder; HAMD, Hamilton Depression Scale; HRS, Hyposmia Rating Scale; PDQ-39, Parkinson Disease Quality of Life Questionnaire-39 item version, LEDD, levodopa equivalent daily dose. The scores of UPDRS items 20 and 21 added up to the tremor score. The score for item 22 was the rigidity score. The scores for items 23 to 26 added up to the bradykinesia score. The scores for items 27 to 30 added up to the postural instability score. Disease motor subtype was classified as tremor-dominant (TD) phenotype when the ratio of tremor score and postural instability and gait difficulty (PIGD) score was no less than 1.5, while patients with a ratio of no more than 1.0 were defined to PIGD phenotype and rest of patients belonged to the indeterminate phenotype.

Supplementary Table 6. Primers used in the present study

Name	Primers for PCR
GBA1 Exon2	F: 5’-TGTGGGCCTTGTCCTAATGA-3’<br>R: 5’-GGTTTCAAAATTCCTCACCCCT-3’
GBA1 Exon4	F: 5’-AACCATTACACCCCTCACCC-3’<br>R: 5’-GGCAGGTATATCATTTGAGGTCA-3’
GBA1 Exon6	F: 5’-CCAAGCTCAAGGTAGGCATTC-3’<br>R: 5’-AGAACAGCCTGGCGAAAC-3’
GBA1 Exon7	F: 5’-GTTTCGCCAGGCTGTTCT-3’<br>R: 5’-CTGGGTTTGCATGAGTGAGC-3’
GBA1 Exon8	F: 5’-GGGGTGGTGGTTCATGTATG-3’<br>R: 5’-TGTGCAGACCTGTGAAGGAA-3’
GBA1 Exon9	F: 5’-GTCACCCAACTCCAGGATT-3’<br>R: 5’-GAACACCTTCCTGCTCCCT-3’
*GBA1*Exon10&Exon11	F: 5’-GACTGGAACCTTGCCCTGA-3’<br>R: 5’-TGCTGTGCCCTCTTTAGTCA-3’
	Primers for Nested PCR
GBA1 Exon1-Exon5	F: 5’-CCTAAAGTTGTCACCCATAC-3’<br>R: 5’-AGCAGACCTACCCTACAGTTT-3’
GBA1 Exon1	F: 5’-CTGTGTCATGTGACGCTCCT-3’<br>R: 5’-CAGTGCCAGGATTCCAGAAG-3’
GBA1 Exon3	F: 5’-ATGTGTCCATTCTCCATGTC-3’<br>R: 5’-GGTGATCACTGACACCATTT-3′
GBA1 Exon5	F: 5’-GCAAGTGATAAGCAGAGTCC-3’<br>R: 5’-AGCAGACCTACCCTACAGTTT-3’
